# Assessment of Morphological, Anatomical and Palynological Variation in the Medicinal Plant *Disporopsis longifolia* Craib (Asparagaceae) for Botanical Quality Control

**DOI:** 10.3390/plants12020259

**Published:** 2023-01-05

**Authors:** Anuwat Sarapan, Trevor R. Hodkinson, Chalermpol Suwanphakdee

**Affiliations:** 1 Department of Botany, Faculty of Science, Kasetsart University, Bangkok 10900, Thailand; 2 Department of Botany, School of Natural Sciences, Trinity College Dublin, The University of Dublin, Dublin D2, Ireland; 3 Palynology Special Research Unit, Department of Botany, Faculty of Science, Kasetsart University, Bangkok 10900, Thailand

**Keywords:** anatomical characters, Asia, lectotypification, medicinal plants, pollen morphology, quality control, species diagnosis, tropical plants

## Abstract

*Disporopsis longifolia* Craib is an Asian medicinal plant belonging to the Asparagaceae family. The plants are well known for their steroidal saponins and phenolic compounds and are traditionally used as tonics for back pain, bellyache, cough, diabetes, asthma, pneumonia and rheumatism. However, they are challenging to identify to species level using morphology. This raises a serious concern for their medicinal applications where botanical quality control is essential. The most appropriate morphological, anatomical and pollen characters for species diagnosis were therefore determined. Synonyms were identified and lectotypification provided. The morphological characters were described from 76 fresh and dried specimens to include a broad range of materials from differing habitats and locations. Paraffin and peeling methods were applied for anatomical studies of leaves and stems and a modified acetolysis method was undertaken for pollen morphology. This paper compares the new character data to published data from other species in the genus, namely *D. aspersa*, *D. fuscopicta*, *D. jinfushanensis*, *D. pernyi* and *D. undulata.* This is the first report of such anatomical and pollen morphology characters for *D. longifolia*. The results provide accurate morphological, anatomical and palynological characters for quality control and are best applied in combination with each other.

## 1. Introduction

Species of the genus *Disporopsis* Hance including *D. longifolia* Craib are used as medicinal plants and are popular in China and several other countries in Southeast Asia such as Laos, Myanmar, Thailand and Vietnam [1,2,3,4]. The rhizome or whole plant is used as a tonic for back pain, bellyache, cough, asthma, pneumonia and rheumatism [1,2,3,4]. Moreover, the rhizome of *D. longifolia* can be eaten with betel nut (*Areca catechu* L., Arecaceae Juss.) and it is an ingredient in chicken soup [5,6].

Some phytochemical studies have been undertaken on the genus that is characterized by its steroidal saponins and phenolic compounds. *Disporopsis aspersa* (Hua) Engl. ex Diels, a close relative of *D. longifolia*, contains the ester (2-isopropyl-5-methylphenyl 2-(naphthalen1-yl) acetate) and furanone (2-C-Methyl-d-threono-1, 4-lactone (2)) which has antifungal, neuritogenic and anti-inflammatory activity [7,8]. Several chemical compounds have been isolated from *D. longifolia* such as a range of known spirostanol glycosides and two new fucose-containing spirostanol glycosides, namely (25*R*)-spirost-5-en-3β-yl *O*-β-d-glucopyranosyl-(1→4)-β-d-fucopyranoside and (25*S*)-spirost-5-en-3β-yl *O*-β-d-glucopyranosyl-(1→4)-β-d-fucopyranoside [9]. However, meaningful interpretation of these phytochemical data is reliant on accurate biological species delimitation. The species of the genus are very similar at a morphological level and they share many common characters. They are perennial erect herbs with moniliform rhizomes and erect and distal arching aerial stems. Their leaves are simple with alternate arrangement and the flowers are solitary or in a raceme. The solitary flower is single or fascicled. The perianth is campanulate and has a corona. The anther has two thecae and is introrse, and the stigma is capitate or slightly trifid. The fruits are berries [10,11]. Because of this morphological similarity, there is a serious issue with species recognition and identification for medical applications.

The genus *Disporopsis* was described and published in 1883 by Hance [12]. It was originally classified in the tribe Polygonateae by Benth and Hook f. [13,14,15,16,17] and placed in Liliaceae Juss. s.l. based on its connate perianth with six lobes and its six stamens that have two locules with longitudinal split [13]. Later, it was transferred to Convallariaceae Horaninow by Conran and Tamura [10] based on the sympodial rhizome and its inflorescence that is a raceme in the axillary leaf. It also has a haploid chromosome number of n = 20 [10,18]. The Convallariaceae was then reduced to tribe Convallariae Engl. in family Asparagaceae Juss. [19,20]. According to phylogenetic analysis using plastid DNA, *Disporopsis* is recognized as monophyletic and grouped with *Convallaria* L., *Heteropolygonatum* Tamura and Ogisu., *Maianthemum* F.H. Wigg., *Smilacina* Desf., and *Polygonatum* Mill. in tribe Polygonateae [15,16,17]. The APG IV also classified *Disporopsis* in tribe Polygonateae in subfamily Nolinoideae Burnett of Asparagaceae [19,20]. The members of this genus are distributed in tropical and subtropical zones in Asia. Seven species are recognized including *D. aspersa* (Hua) Engl, *D. bodinieri* (H.Lév.) Floden, *D. fuscopicta* Hance, *D. jinfushanensis* Z.Y. Liu, *D. longifolia* Craib, *D. pernyi* (Hua) Diels and *D. undulata* Tamura and Ogisu [11,21,22,23].

*Disporopsis longifolia* is very commonly distributed in Northern and Northeastern floristic regions of Thailand and its rhizomes are used for food and traditional medicine [7,24]. This paper aimed to use herbarium specimen label information to gather location, phenological and ethnobotanical data. It then determined the most useful morphological, anatomical and palynological characters for species diagnosis to provide the necessary data for quality control in the world’s medicinal plant market. To achieve this, studies were undertaken on 76 samples from fresh and herbarium material. The results from *D. longifolia* are compared with other species in the genus and related genera, using previously published studies, that could potentially be confused with the target species for medical application.

## 2. Results

### 2.1. Species Description and Morphological Characters

**Figure 1:**
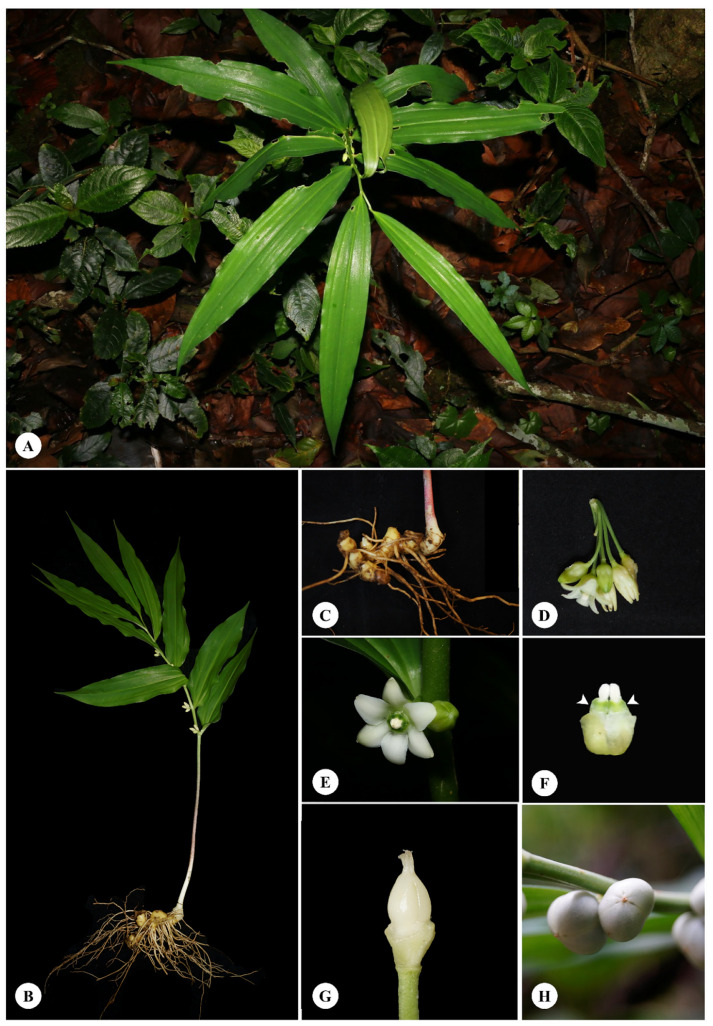
Morphological features of *Disporopsis longifolia*. (**A**) Habit; (**B**) whole plant; (**C**) moniliform rhizome; (**D**) fascicle flowers; (**E**) flowers; (**F**) stamen and corona (arrowheads); (**G**) pistil; (**H**) fruits. Specimen: Chiang Mai, *A. Sarapan 41*. Photos by A. Sarapan.

Morphological data were recorded from 76 plants (listed in Materials and Methods) and summarized into the following description for *Disporopsis longifolia*: Type material in Kew (K; holotype), Edinburgh (E; isotype) and Paris (P; lectotypes) herbaria were viewed (! notation below) and protologues consulted. This description can be used with the photographs in Figure 1 to accurately identify the species, and it follows the format and notation from the Flora of Thailand Project [25]. Synonyms are listed and lectotypification provided.

*Disporopsis longifolia* Craib, Bull. Misc. Inform. Kew 10: 410. 1912; Charoenphol, Thai For. Bull. (Bot.) 7: 90. 1973; Songyum and Tamura, Fl. China 24: 232. 2000. Type: Thailand, Chiang Mai, Doi Suthep National Park, *Kerr 687a* (holotype K, photo!; isotype E, photo!).

Synonyms:

*—Polygonatum laoticum* Gagnep., Bull. Soc. Bot. France 81: 288. 1934. Type: Laos, Paklai à Luang Prabang, *C. Thorel 3319* (lectotype, P (P00687140 (photo!)), isolectotypes, P

(P00687141 (photo!)), (P00687142 (photo!))).

*—Polygonatum tonkinense* Gagnep., Bull. Soc. Bot. France 81: 288. 1934. Type: Vietnam, Tonkin, Tuyen-quang, Bavi Mountain, *B. Balansa 4146* [lectotype, P (P00038269 (photo!)), isolectotype, P (P00687139 (photo!))].

Perennial erect herb; *rhizomes* moniliform; *aerial stems* erect and distally arching, 50–150 cm long, glabrous; lower stem rounded and shallow grooves, green or dark purple to purplish red and with purple maculate; distally stem rounded to rectangular, two-ridged, green. *Leaves* simple, alternate or subspiral, lanceolate to elliptic, 24–27 × 4–10 cm, base cuneate, margin entire, apex acuminate or caudate, green, glabrous, coriaceous or papery when dried, 3–5 veins, palmately netted venation; petiole 0.5–1 cm long, wings, green and light green, glabrous. *Flowers* single or in fascicle of 2–7 flowers, rotate, 1–1.3 cm long, perianth connate to tube and 6 lobes, white; lobe ovate, two-whorled, 0.6–0.8 × 0.4–0.5 cm, margin entire, apex obtuse with cilia, 1–3 keels, white. *Corona* opposite lobe of perianth tube, apex emarginate, white or green. *Stamens* six inserted on lobe apex of corona, 0.2–0.3 cm long; anther oblong, two-thecae, dorsifixed, longitudinal split, introrse; filament cylindric shaped, 1–1.5 cm long, white or yellow, glabrous. *Ovary* globose, 0.4–0.5 × 0.2–0.3 cm, three-locules, 3–5 ovules, axile placentation; style cylindric-shaped, 0.1–0.2 cm long, 3-grooves, white, glabrous; stigma capitate. *Fruits* berry, globose, 0.5–0.7 × 0.5–0.8 cm, three-groove, apex with mucro, glabrous, pendulous, green, white, pearl or back. *Seed* 1–3, globose, light brown or brown, smooth surface (Figure 1).

*Disporopsis longifolia* is distinguished by its moniliform rhizome and the flowers that are single or in a fascicle with 2–7 flowers. The flowers are campanulate and white. The perianth has a corona and stamens are inserted on the corona lobe. The stigma is capitate and the fruits are green and white when ripening. According to herbarium labels, the rhizome is used as a tonic for cystitis, diuretics, cough, headaches, cold and fever, and the leaves are used to treat knee pain. It is distributed in China, Myanmar, Thailand, Laos and Vietnam and situated near streams or on limestone areas of evergreen forest at 300–1700 m in altitude. It flowers in May to July and fruits in July to February. Its vernacular names include Chua Mo Hua Cho, and Lo Ma Chor Ju.

Lectotypification notes: *Polygonatum laoticum* Gagnep. and *P. tonkinense* Gagnep. were synonymized under *Disporopsis longifolia* Craib by Sungyun and Tamura [11] but they did not list the type. The type *Polygonatum laoticum* Gagnep. is *C. Thorel 3319*. Later, we found three herbarium sheets in P. We selected P00687140 as the lectotype, and selected P00687141 and P00687142 as isolectotypes. *Polygonatum tonkinense* was simultaneously published and the types are *A.J.B.Chevalier 37,746*, *B. Balansa 4145* and *B. Balansa 4146* in protologue. We found four sheets in P, namely *A.J.B. Chevalier 37,746* (P00687136), *B. Balansa 4145* (P00687137), *B. Balansa 4146* (P00038269) and *B. Balansa 4146* (P00687139). We selected *B. Balansa 4146* (P00038269) as the lectotype and *B. Balansa 4146* (P00687139) as the isolectotype.

### 2.2. Stem, Petiole and Leaf Anatomy

**Figure 2:**
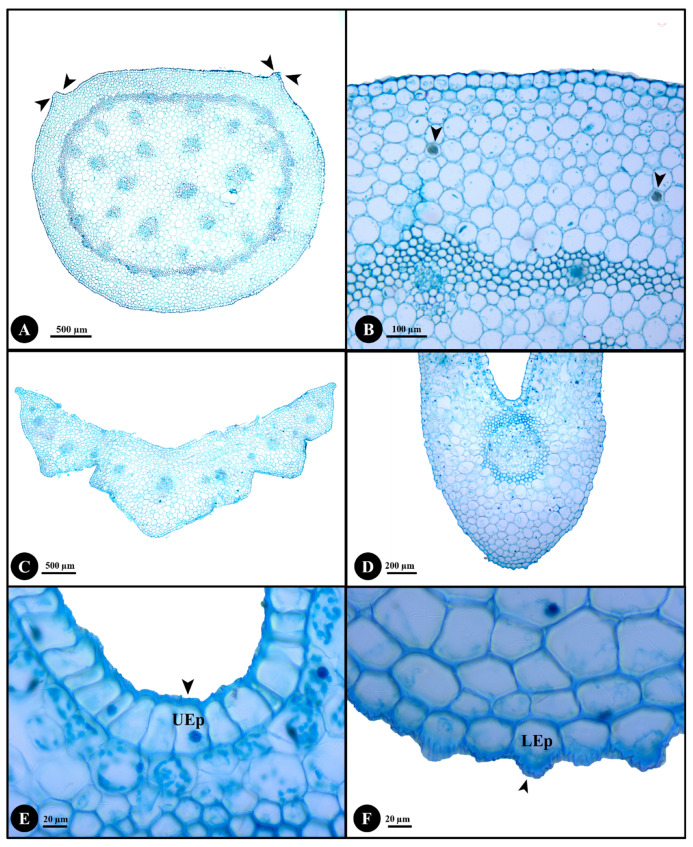
Anatomical characters of stem, petiole and midrib of *Disporopsis longifolia*. (**A**) Stem with ridges (arrowheads); (**B**) raphides (arrowheads); (**C**) petiole; (**D**) midrib; (**E**) echinate cuticle thickening (arrowhead) of upper epidermis; (**F**) echinate cuticle thickening (arrowhead) of lower epidermis; (Abbreviations: UEp, upper epidermis; LEp, lower epidermis).

**Figure 3:**
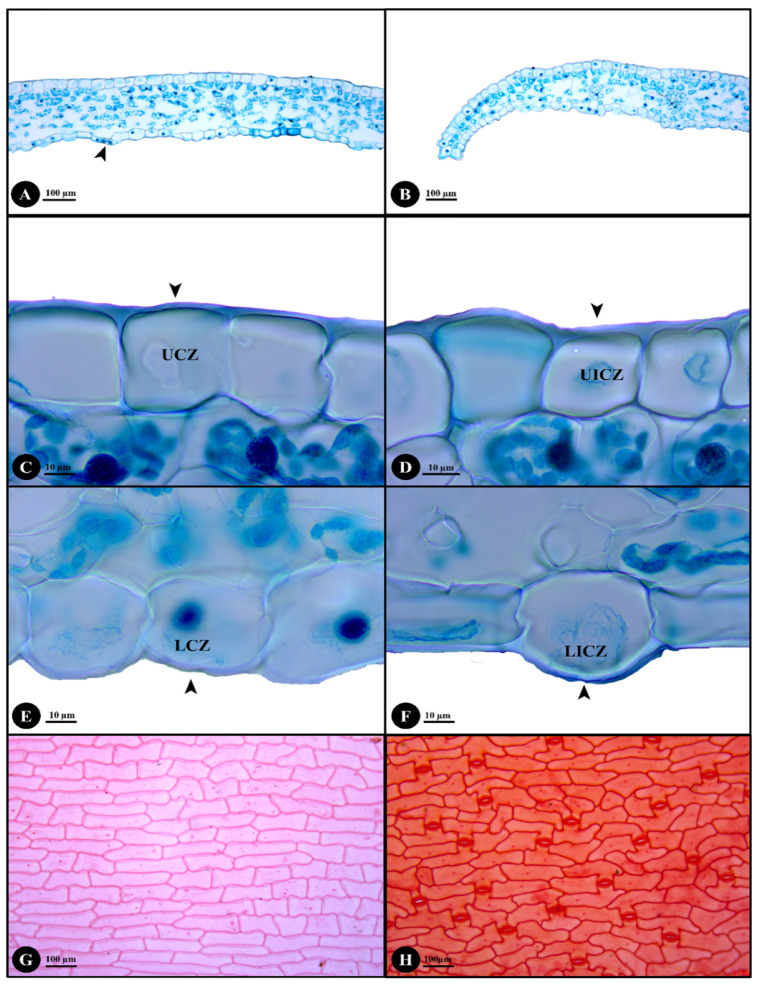
Leaf anatomical characters of *Disporopsis longifolia*. (**A**) Raised stomata (arrowheads); (**B**) revolute margin; (**C**) smooth cuticle thickening (arrowhead) of upper epidermis at coastal zone; (**D**) smooth cuticle thickening (arrowhead) of upper epidermis at intercostal zone; (**E**) smooth cuticle thickening (arrowhead) of lower epidermis at coastal zone; (**F**) smooth cuticle thickening (arrowhead) of lower epidermis at intercostal zone; (**G**) upper epidermis surface; (**H**) lower epidermis surface; abbreviations: UCZ, upper epidermis in coastal zone; UICZ lower epidermis in coastal zone; LCZ, lower epidermis in coastal zone; LICZ, lower epidermis in intercostal zone.

The stem, petiole, leaf (including midrib, margin, costal and intercostal zones) and leaf surface results are shown in Figure 2 and Figure 3.

*Stem anatomy:* The stem is rounded with two small ridges (Figure 2A). The epidermis is rounded or rectangular with echinate cuticle thickening. The cortical region comprises 7–13 layers of parenchyma cells with air spaces and raphides (Figure 2B). The parenchyma cells are rounded, subrounded or rectangular and there is intercellular space. Pericycular vascular bundles and ground tissue vascular bundles are present; both are the closed-collateral bundle type. The pericycular vascular bundle regions have 28–36 bundles with 3–7 layers of sclerenchyma cells. The sclerenchyma cells form rings and are surrounded with pericycular vascular bundles. The ground tissue has parenchyma cells, intercellular space, air spaces and vascular bundles. The vascular region in the ground tissue has 15–28 bundles with sclerenchyma cells surrounding the bundles.

*Petiole anatomy:* The petiole is V- or U-shaped (Figure 2C). The upper and lower epidermis cells have echinate cuticle thickening and papillae that are present only on the lower epidermis. The ground tissue includes parenchyma and collenchyma. The parenchyma cells are rounded, subrounded or polygonal with intercellular space. The collenchyma cells are rounded or subrounded. The vascular bundle regions distributed in the ground tissue have 11–14 bundles that are of the closed-collateral type with sclerenchyma surrounding them.

*Lamina anatomy:* The lamina comprises midrib, margin, costal and intercostal zones. The midrib is U-shaped (Figure 2D). The epidermal cells of both leaf surfaces are rounded, subrounded or rectangular with echinate cuticle thickening (Figure 2E,F). The ground tissues have only rounded or subrounded shaped parenchyma cells. The vascular bundles distributed in the ground tissue are closed-collateral bundle type with sclerenchyma surrounding them. The margin is revolute with large epidermal cells (Figure 3B). The echinate cuticle is thickened on the upper and lower epidermal cells and papillae are present.

The leaf is hypostomatic with raised stomata (Figure 3A). The coastal zone of the upper and lower epidermis has smooth cuticle thickening (Figure 3C,E) and the cells are rounded, square or elongated rectangular. The intercostal zone of upper and lower epidermis also has smooth cuticle thickening (Figure 3D,F) and the cells are also rounded, square or elongated rectangular. The palisade mesophyll is columnar or subrounded with 1–2 layers. The spongy mesophyll is rounded or irregular. The leaf surface of upper and lower epidermis has elongated rectangular and polygonal cells (Figure 3G,H). The anticlinal cell wall is strongly straight and the periclinal cell wall is straight to slightly undulate. The lower epidermis has anomocytic stomata (Figure 3H).

### 2.3. Pollen Morphology

**Figure 4:**
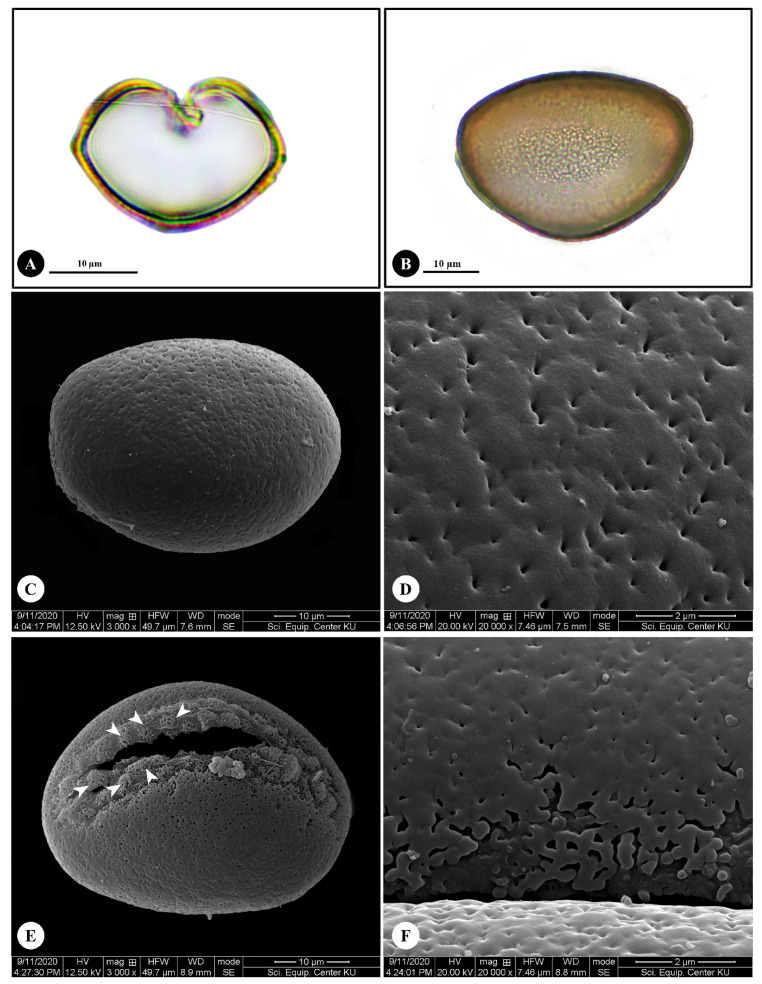
Pollen morphology of *Disporopsis longifolia*. (**A**,**B**) Light microscope images; (**A**) polar view; (**B**) equatorial view. (**C**–**F**) Scanning electron microscope images; (**C**) equatorial view; (**D**) perforate ornamentation; (**E**) monosulcate and areolate ornamentation (arrowheads); (**F**) areolate ornamentation (top view).

The pollen of *Disporopsis longifolia* are heteropolar monads (Figure 4A,B) and the grains have bilateral symmetry. The pollen is medium size with polar axis (P) 40–42 ± 0.79 μm in length, and equatorial axis (E) 29–31 ± 0.73 μm in length. The shape is prolate (P/E = 1.33–1.44 ± 0.04 μm) (Figure 4B,C) and the grain is monosulcate (Figure 4E). The exine thickening is 1–2 ± 0.43 μm in depth. The ornamentation is usually perforate (Figure 4D) and areolate at aperture areas (Figure 4E,F).

## 3. Discussion

Species of the genus *Disporopsis* are used as medicinal plants for many applications [1,2,3,4,5,6,7,8]. Our studies using information from the literature and herbarium labels indicates that the rhizome of *D. longifolia* was used for traditional medicine by boiling it three times and the last water used as a tonic for cystitis, diuretics, cough, headaches, cold and fever. Moreover, the leaves are used as medicine to treat knee pain. In the northern parts of Thailand, the rhizome of *D. longifolia* is used for eating with betel nut [7].

This paper studied a wide range of samples across its natural distribution and has been able to describe key characteristics of this plant based on gross morphology, anatomy and pollen morphology. It is possible, but difficult, to identify the species using the morphological characters described here, especially if flowering material is unavailable. Therefore, it is advisable to include anatomical and pollen morphological studies to confirm taxonomic identity. In summary, based on previous studies [11,23,24] and observations in this paper, *D. longifolia* has underground moniliform rhizomes and its aerial stem is erect to distally arching. The flowers are solitary or fascicled with 2–7 florets in the axil of a leaf. The flowers are of campanulate shape and white and the perianth is connate with six lobes. The tepal apex is ciliate. The stamens are introrse and longitudinally split and they are inserted on the perianth lobes. The stigma is capitate and the fruits are green to white berries.

Care must also be taken not to confuse *Disporopsis* with its close relative *Polygonatum*, both in tribe Polygonateae [19,20,26], because they have similar morphology. They share moniliform rhizomes and the leaves have alternate arrangement. The flowers are cylindrical to campanulate and are in axils of leaves, and the fruits of both are berries [10,20,26]. Both genera have been used as medicinal plants in Thailand [7,8] as whole plants or especially rhizome [1,2,3,4,5,6,7,8]. Therefore, it is important to distinguish the two correctly. A corona is present in *Disporopsis* but absent in *Polygonatum*. The filament of *Disporopsis* is inserted on the corona but in *Polygonatum*, it is inserted on the perianth.

Charoenphol [24] reported *D. longifolia* in Thailand and described the flower characters with fascicles of 5–6 florets or more. Our study determined that the flowers are solitary to fascicled with 2–7 florets in the same area and populations. Therefore, this paper confirmed that the *D. longifolia* has solitary flowers or fascicled inflorescences with 2–7 florets. In addition, variation in the colour of the lower stem was recorded, which is green, purplish red or green with purple maculate in Thai *D. longifolia* depending on habitat. The number of florets per fascicle has been used for species identification and distinction by others. For example, Songyun and Tamura [11] distinguished *D. longifolia* by its fascicles with 5–10 florets compared to *D. aspersa*, *D. fuscopicta*, *D. jinfushanensis*, *D. pernyi* and *D. undulata* which have solitary flowers or fascicles with 2–3 florets. In this paper, the number of flowers per fascicled inflorescence shows high variability and it is recommended that this characteristic should be avoided for species identification.

Species of *Disporopsis* can be divided into two groups. One group has moniliform rhizomes, including *D. longifolia* and *D. fuscopicta*, and the other has terete rhizomes, including *D. aspersa*, *D. jinfushanensis*, *D. pernyi* and *D. undulata*. The first group, *D. longifolia* and *D. fuscopicta,* also have sagittate anthers [11,12,23] but different corona and fruit. In *D. longifolia*, the corona is fleshy and the lobes do not exceed the anthers. This differs in *D. fuscopicta*, where the corona is membranous and the lobes exceed the anthers. The berry fruits are white in *D. longifolia* or purplish in *D. fuscopicta* [27]. Therefore, the diagnostic characteristics of *D. longifolia* are the moniliform rhizomes, the fleshy corona, the corona lobes not exceeding anthers, and the white berries.

The anatomy of stem, petiole, midrib, leaf margin and leaf surfaces of *D. longifolia* were investigated to detect additional characters for identification. Both *D. fuscopicta* and *D. longifolia* have hypostomatic leaves [27] but they differ by the anticlinal cell walls. The anticlinal cell walls are sinuous in *D. fuscopicta* [27] whereas strongly straight in *D. longifolia*. Therefore, the anticlinal cell walls are significant characters for species identification. Some samples were collected from limestone areas and were determined to have raphides in their stems, whereas some samples collected from other locations such as near streams, on mountain slopes or on mountain summit in evergreen forest lacked raphides. Therefore, raphides in stem are a plastic characteristic that varies based on locality and they cannot be used reliably for species delimitation.

Anatomical characters of genus *Disporopsis* are poorly known and no anatomical studies of *D. longifolia* have previously been published. In summary, the stem of *D. longifolia* has two small ridges, the epidermal cells of stem, petiole, midrib and leaf margin all have echinate cuticle thickening, and the upper and lower epidermis in costal and intercostal zones have smooth cuticle thickening. Papillae are present on the lower epidermis of the petiole and leaf margin. The cortical region has 7–13 layers of parenchyma cells that sometimes accumulate raphides. There are pericycular vascular bundles and vascular bundles in the ground tissue, and the bundles in stem, petiole and leaves are of the closed-collateral bundle type. The petiole is V- or U-shaped and midrib is U-shaped. The petiole has 11–14 vascular bundles. Raised stomata are present on hypostomatic leaves. The palisade mesophyll is columnar or subrounded with 1–2 layers. The spongy mesophyll is rounded or irregular. The leaf margin is revolute and has large epidermal cells. In addition, the upper and lower epidermis of the leaf have elongated rectangular and polygonal cells. The anticlinal cell wall is strongly straight and periclinal cell wall is straight to slightly undulate. The lower epidermis is of anomocytic stomata type.

Pollen morphological characters of *Disporopsis* are also poorly documented and this paper is the first report on pollen morphological characters of *D. longifolia*. Pollen grains of *D. longifolia* are heteropolar monads of medium size, have bilateral symmetry, and are prolate and monosulcate. The ornamentation is perforate and areolate at the aperture areas. Pollen morphologies have been described for *D. aspersa*, *D. fuscopicta* and *D. pernyi* in China [28]. They shared common characters including the monads, bilateral symmetry, monosulcate aperture and reticulate ornamentation. In general, it was determined that the pollen morphological evidence did not strongly support species identification [28]. In contrast, it was determined that the *D. longifolia* has perforate ornamentation in general areas and areolate ornamentation in the aperture area. These two types of pollen ornamentation, perforate and areolate, are useful to distinguish *D. longifolia* from the aforementioned species. In addition, Wang et al. [28] reported medium pollen size of *D. aspersa*, *D. fuscopicta* and *D. pernyi*, including polar axis of 20.3–25.9 μm in length and equatorial axis of 41.4–44.3 μm in length. In contrast, *D. longifolia* had a longer polar axis of 40–42 ± 0.79 μm and shorter equatorial axis of 29–31 ± 0.73 μm. The differences are again significant for taxonomic separation of *D. longifolia* from *D. aspersa*, *D. fuscopicta* and *D. pernyi*.

Given the potential value of *D. longifolia* and its use in traditional medicine, it is important to conserve its wild populations for future utilization. No IUCN (Standard and Petitions Committee 2019) conservation status category had previously been proposed for this species, so we here designate the status of Least Concern (LC). This is because *Disporopsis longiflolia* has a wide distribution in China, South-Eastern Asia and in Thailand, and it is distributed in Northern and North-Eastern protected areas.

Our studies have provided essential baseline data for future phytochemical assessments where accurate species identification is essential. We have also provided essential lectotypification of synonyms. There are several other methods available for botanical quality control of medicinal plants and including molecular DNA barcoding and chemical characterisation [29,30], but these studies are not always the most appropriate and they rely on accurate species identification using morphology and anatomy in the first instance.

## 4. Materials and Methods

**Figure 5:**
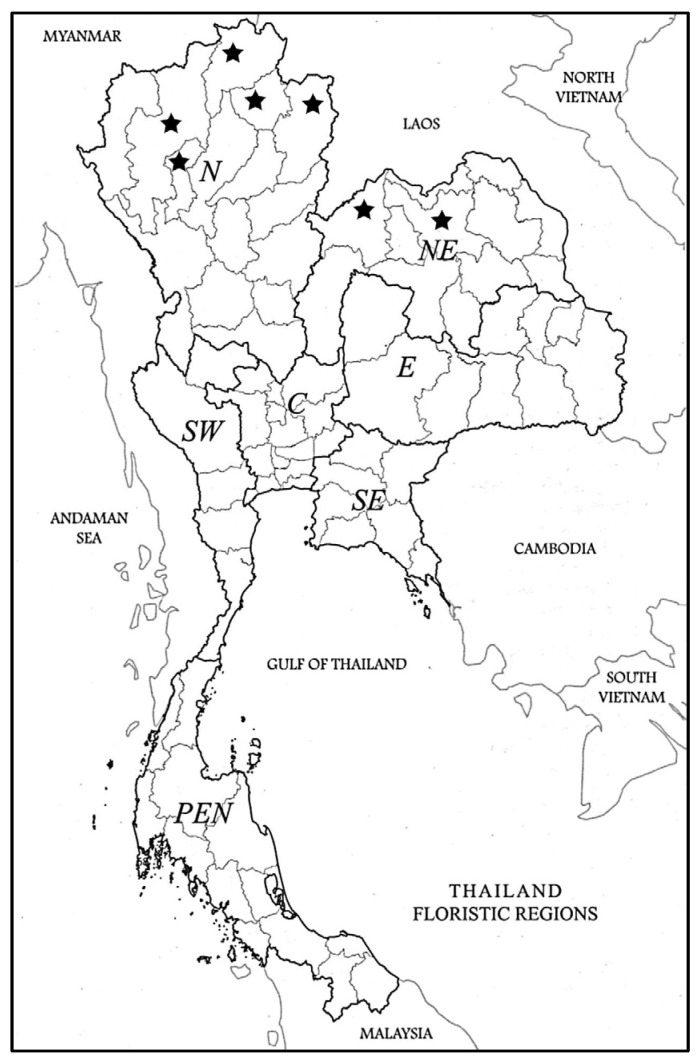
The distribution of *Disporopsis longifolia* in Thailand and the sample locations. *D. longifolia* is only located in the Northern (N) and North-Eastern (NE) Floristic Regions. Floristic regions defined in [31]. It is also found in China, Myanmar, Laos and Vietnam.

The morphological characters were described from fresh and dried specimens as listed under specimens examined below. The fresh specimens were collected from 10 field sites in the Northern and North-Eastern Floristic Regions of Thailand [31], Figure 5. Voucher specimens were prepared and deposited in BK, BKF, KKU and QBG (acronyms of herbaria follow Thiers [32] Index Herbariorum). Herbarium specimens were consulted from BK, BKF, KKU, QBG and the online herbarium databases including BM, C, E, G, K, K-W, L, P, S, W and WU to gain additional samples.

### Specimens Examined

Note: 76 specimens were examined; location and date of collection, collector name, collection number, and herbarium code are indicated for each specimen below in the Northern and North-Eastern floristic regions.

NORTHERN: Chiang Rai (Doi Luang National Park, 27 October 2015, Norsaengsri 12,482 (QBG); 23 September 2015, Tanming 928 (QBG)); Chiang Mai (Ban Huai Chay, Mae Tha subdistrict, Mae On district, 5 June 2021, Sarapan 41 (BK, BKF, KKU, QBG); Ban Mae Sa Mai, Mae Rim, 4 August 2012, Nguanchoo 472 (QBG); Banphanokkok, Pong Yaeng, 24 December 2007, Jutupol 07-146 (QBG); Doi Chiangdao Wildlife Research Station, Chiangdao, 8 February 2021, Sarapan 36 (BK, BKF, KKU, QBG); Doi Hua Suean, Doi Doi Inthanon National Park, 6 July 2019, Sarapan 35 (BK, BKF, KKU, QBG); Doi Inthanon National Park, Chom Thong, 15 July 1922, Kerr 6361(BK); 30 January 1996, Nanakorn et al., 5944 (QBG); 17 June 2019, Sarapan 20 (BK, BKF, KKU, QBG); Doi Pha Hom Pok National Park, 9 February 2021, Sarapan 38 (BK, BKF, KKU, QBG); Doi Suthep, 16 September 1999, Suksathan and Middleton 1831 (QBG); 4 July 2020, Sarapan 28 (BK, BKF, KKU, QBG); Highland QBG, Mae Rim, 21 June 2012, Nguanchoo 257 (QBG); Huei Mae Mae, 16 July 1998, Pongamornkul 202 (QBG); Mae Rim to Samoeng Road, 23 July 2002, Glamwaewwong 271 (QBG); Mae Sa Mai, Mae Rim, 21 June 2012, Nguanchoo 190 (QBG); Mon Lhong, 20 May 1998, Pongamornkul 98 (QBG); 6 June 2021, Sarapan 43 (BK, BKF, KKU, QBG); Nam Tok Nang Koi, Samoeng, 24 September 1997, Nanakorn et al., 9647 (QBG); Siribhume Waterfall, 29 November 2019, Sarapan 17 (BK, BKF, KKU, QBG)); Phayao (Jum Pha Thong waterfall, Doi Luang National Park, 5 November 2015, Muangyen 342 (QBG); 25 July 2016, Muangyen 1235 (QBG); Pong, 1 July 2009, Pongamornkul 2701 (QBG); Ban Huak, Phu Sang district, 24 June 2014, La-ongsri, Panyanchan and Tatiya 3405 (QBG); 27 June 2014, La-ongsri, Panyanchan and Tatiya 3511 (QBG); Tham Pra Daeng, Rom Yen Subdistrict, Chiang Kham, 2 May 2013, La-ongsri et al., 2899 (QBG); 18 June 2013, La-ongsri et al., 2936 (QBG); 29 August 2018, Sonsupab et al., sn (BK)); Nan (Ban Huay York, Wiang Sa district, 22 July 2016, Nguanchoo 808 (QBG); Ban Num Tuang, Mae Charim district, 27 December 2016, Nguanchoo 1013 (QBG); Ban Rom Klaw, Mae Charim district, 18 February 2016, Nguanchoo 606 (QBG); Doi Phu Kha National Park, 2 July 1999, Srisaga, Watthana and La-ongsri 827 (QBG); 3 December 1999, Srisaga 1196 (QBG); Sakoen National Park, Yod, Song Kwae, 15 December 2010, La-ongsri and Romkham 1211 (QBG); Tham Pra Khaw, Doi Phu Kha National Park, Pua, 5 July 2001, Srisaga 1931 (QBG); Tham Pra Kong, Doi Phu Kha National Park, Pua, 26 June 2002, Srisaga 2514 (QBG); 26 June 2002, Srisaga 2517 (BKF, QBG)); Lumphun (Doi Khun Tan National Park, 12 August 2020, Sarapan 32 (BK, BKF, KKU, QBG)).

NORTH-EASTERN: Loei (Na Haew, 9, December 1996, Nanakorn et al., 8003 (QBG); 10 December 1996, Nanakorn et al., 8112 (QBG); Phu Kradueng National Park, 10 June 2013, Suddee 4484 (BKF); 28 May 2018, Sarapan 4 (BK, BKF, KKU, QBG); Route Head Quaters to TKl the Hua Hom, Phu Suan Sai National Park, Maknoi 2743 (QBG); 15 May 2008, Maknoi and Srisanga 2246 (QBG); Tad Hueang Waterfall, Phu Suan Sai National Park, Na Haew, 16 May 2008, Maknoi and Srisanga 2323 (QBG); 8 July 2008, Maknoi 2452 (QBG); Udon Thani (Huai Ei Hang, Nam Som, 9 December 2009, Boonprakop 133 (QBG).

Ethnobotanical data were collected from label information on herbarium sheets, the literature reviews and interviews with local tribesman and local people of the Karen ethnolinguistic group (Sino-Tibetan language) also known as the Kayin, Kariang or Kawthoolese.

Anatomical characters were studied using the paraffin method following Thammathaworn [33] and permanent slides of stem, petiole and leaf were prepared following Kermanee [34]. The permanent slides were prepared using the paraffin method. Fresh stem and leaves were fixed in 50% formalin acetic acid (FAA) for 24 h and then washed in 50% ethanol. The samples were dehydrated in a graded Tertiary Butyl Alcohol (TBA) series (50, 70, 85, 95 and 100%, respectively). In addition, samples were immersed in a mixture of pure TBA and liquid paraffin at 60 °C for 12 h and embedded in paraffin at 60 °C. The samples in paraffin blocks were sectioned with 15–25 μm thickness using a rotary microtome (Leica RM2165, Germany). The sections were affixed on slides. These slides were stained with a combination of safranin O and fast green. The stained samples were washed with 95% ethanol and dehydrated with absolute ethanol. The samples were cleared continuously in xylene and absolute ethanol (1:1) and immersed in pure xylene. The slides were mounted with DPex and investigated under a light microscope (Carl Zeiss Primo Star).

Permanent slides of the epidermis were prepared using a peeling method [34]. The method is applicable to fresh and spirit preserved leaves. The epidermis was scraped off with a razor blade and washed with water. Then, it was soaked in 0.1% chlorine, washed with water and stained with 1% safranin for 3–5 min. Samples were then dehydrated by ethanol series (30%, 50%, 70%, and 95% respectively) and cleared with xylene. Finally, the samples were mounted on slides with DPex and photographed under a light microscope (Carl Zeiss Primo Star).

A modified acetolysis technique was applied for the preparation of the pollen morphological characters [35] and the pollen slides were investigated under a light microscope (Carl Zeiss Primo Star) and a scanning electron microscope (FEI, Quanta 450) following Halbritter et al. [36]. The anatomical and pollen permanent slides were stored at the Palynology Special Research Unit, Department of Botany, Faculty of Science, Kasetsart University, Bangkok.

## 5. Conclusions

The detailed data and descriptions presented here allow for accurate identification of *D. longifolia*. These characters are best used in combination and can be applied to living and herbarium material and material with different parts missing. The anatomical and palynological characteristics are reported here for the first time. DNA barcoding and chemical characterisation can also be used for species identification, but these techniques rely on accurate baseline species identification using morphology and anatomy in the first instance. Our study therefore provides the essential information required for further molecular and phytochemical evaluation where species identification is of paramount importance.

## Data Availability

Data sharing not applicable. No new data were created or analyzed in this study. Data sharing is not applicable to this article.
